# “It doesn’t Always Have to Be an Expert”: Professionals’ Perceptions of Practical Aspects of the Existential Dimension of Care for People Approaching Death

**DOI:** 10.1177/00302228241272637

**Published:** 2024-08-10

**Authors:** Carl Bäckersten, Ulla Molander, Inger Benkel, Stina Nyblom

**Affiliations:** 1 Palliative Centre, 56749 Sahlgrenska University Hospital, Gothenburg, Sweden; 2 Sahlgrenska Academy, Institute of Medicine, 70712 University of Gothenburg, Goteborg, Sweden

**Keywords:** existential care, spiritual care, healthcare professionals, end-of-life, palliative care

## Abstract

‘Existential’ can be seen as a broad term for issues surrounding people’s experiences and way of thinking about life. This study examined availability of existential care and found that many different staff categories performed existential care. Existential care is associated with conversations and experienced as both easy and difficult; several factors were cited, e.g. insufficient time, stress and the difficulty of addressing existential questions for oneself. Respondents reported need for education, guidance and reflection around existential issues and care. Existential care is described as a natural part of patient care that all professional categories have a responsibility to offer.

## Introduction

Existential experiences can often come to the fore when a person or their loved ones are suffering from a severe and/or life-threatening disease. This can evoke a variety of thoughts and emotions, such as anxiety about death, hope and hopelessness, meaning and loss of freedom, and often relates to relationships, social connections, isolation and loneliness (Benkel & Molander, 2017; Bolmsjö et al., 2019; Chen et al., 2022; King & Hicks, 2021; Sjöberg et al., 2022). The existential dimensions and associated questions derive largely from philosophers (e.g. Kierkegaard, Nietzche and Heidegger) who have developed thoughts over the centuries. The existential philosophy tradition includes both theistic and secular worldviews and it has been argued that fundamental values and beliefs should have a common label (Breitbart, 2007).

In palliative care, the inclusion of the spiritual/existential dimension is considered important to achieve holistic care (Richardson, 2012). The European Association for Palliative Care (EAPC) reference group for spiritual care has addressed this question and provided the following definition of ‘spirituality’: *Spirituality is the dynamic dimension of human life that relates to the way persons (individual and community) experience, express and/or seek meaning, purpose and transcendence, and the way they connect to the moment, to self, to others, to nature, to the significant and/or the sacred* (Nolan et al., 2011).

Although the Scandinavian countries are often considered highly secular, there is an increased interest and attention to the spiritual/existential dimension of care and the subject is sought to be approached in new ways (Bäckersten et al., 2023). In accordance with the post-secular perspective, a modern and “enlightened” society does not automatically mean an absence of spirituality and religiosity (Habermas, 2006, 2008).

According to Nissen et al. (2021), spiritual care can be understood as trying to comprehend a patient’s existential orientation, needs and resources in religious, spiritual and secular domains (Hvidt et al., 2022; Nissen et al., 2021). All these three existential domains are situated in a cultural context and influenced by, for example, family, social, historic, economic and educational factors (Hvidt et al., 2022; Nissen et al., 2021; Walach, 2017).

‘Spiritual’ is perhaps the term most commonly associated with spiritual and existential concerns. ‘Existential’, on the other hand, can be seen as a broad term for issues surrounding people’s experiences and perspectives on life; in Sweden, it is often used in healthcare settings and is commonly argued to be the term best suited for these matters in a healthcare context (Browall et al., 2010; Salander, 2006; Sjöberg et al., 2022; Udo et al., 2014).

Attitudes towards the inclusion of existential dimensions in care in Sweden have been investigated in previous research (Browall et al., 2010; Strang et al., 2002). Several studies describe various educational approaches to existential dimensions of care, mainly for nurses. These include communication about existential issues in the form of lectures and reflected discussions. These studies reported that focus group discussions provide a deeper understanding of existential issues and offer autonomy for dealing with existential questions (Alavi & Hosseini, 2019; Ozanne et al., 2022; Strang et al., 2014; Udo et al., 2014).

However, the views of healthcare professionals on the practical application of existential dimensions of end-of-life care in a highly secular context needs further exploration. This study is part of a larger project with an overall aim to investigate how professionals caring for people approaching death perceive the existential dimension of care (Bäckersten et al., 2023). The specific aim of this study was to investigate how professionals perceive the practical aspects of the existential dimension of care in their current workplace.

The research questions were: Do existential needs and existential care occur in your workplace? Who is responsible for the existential care offered and who performs existential care at your workplace? How do you experience talking about existential questions? Do you assess existential needs and care? Do you have any written material to support your work with existential questions/needs/care? Do you need education, guidance and reflection in your work with existential questions/needs/care?

## Methods

A mixed method design including a digital survey with open- and closed-ended questions was used (Tashakkori & Teddlie, 2010). Due to a lack of suitable precursors, a questionnaire was developed by the researchers that contained 27 questions in Swedish (19 main questions with 8 supplementary questions) plus 7 demographic questions. The Swedish word ‘existential’ was used in the questions and was intended to encompass spiritual, religious and secular connotations of an existential nature. To meet the study’s objective, questions were asked about existential issues, needs and care. The questionnaire was pilot tested on healthcare professionals and minor adjustments were subsequently made.

### Data Collection

The survey was sent by mail to 38 managers of healthcare professionals at specialized palliative care units (P), an oncology clinic (O), a municipal (M) healthcare provider of home care and within a nursing home in western Sweden. Units were selected that provide end-of-life care for people with life-threatening illnesses and/or in old age.

If the managers agreed to the unit’s participation in the study, they were asked to forward the questionnaire by email to all staff at the unit who were involved in the provision of care, regardless of whether the person was subordinate to the manager. Information on the number of emails sent by managers was not requested and the response rate therefore cannot be estimated. By answering the survey, the respondents gave their consent.

Data were collected between October 2021 and April 2022.

### Data Analysis

Descriptive statistics were applied to closed-ended questions. For open-ended questions, a qualitative descriptive approach was used to obtain a rich description of professionals’ perceptions of the existential dimension of care (Bradshaw et al., 2017; Neergaard et al., 2009). All researchers read the open-ended questions independently so that a picture could be obtained of common opinions among the participants. All individual authors analysed the data to identify phrases and important features. The most common findings were described and compared against each other and supporting quotes were linked. A joint analysis was then conducted by the authors to provide as objective an interpretation as possible. Differences were discussed until a common conclusion was reached.

## Results

A total of 77 persons answered the survey. The respondents included registered nurses, assistant nurses, physicians and a social worker. Their ages varied from 20 to 65 years and the majority of the participants had been employed between 11 and 30 years. For more details, see Table 1.

**Table 1.** Characteristics of Study Participants (*n* = 77).

Gender	
Female	70
Male	7
Age (years)	
20–30	8
31–40	12
41–50	19
51–60	26
61--	11
No answer	1
Occupational category	
Registered nurse	43
Assistant nurse	18
Physician	11
Social worker	1
Other	4
Years in the profession	
1–5	12
6–10	12
11–20	21
21–30	18
31-	13
No answer	1
Form of care	
Oncology clinic	35
Specialized palliative care	27
Municipal care	15

The results are presented under the following six headings:

Professionals’ experiences of existential needs and care in their workplace; Responsibility to offer and perform existential care in the workplace; Dialogue on existential issues; Assessment of existential needs and care; Written material to support work with existential issues and care; and Need for support – education, guidance and reflection.

### Professionals’ Experiences of Existential Needs and Care in Their Workplace

The overwhelming majority of participants (95%) perceived patients to have existential needs.

Existential needs were often considered an integral part of an individual and could not be attributed to a separate dimension.

“It can never be isolated from other symptoms. There is often an existential dimension to the conversations.”(O)

However, many staff felt that there was too little time and space to address existential needs once they were identified. Participants felt that existential needs could not be met or that existential care was overlooked due to insufficient time and the prioritisation of nursing and medical measures.

“Many patients and relatives wish to talk about existential issues but refrain from bringing them up as staff seem stressed and unreceptive.”(P)

“Too often there is a lack of time.”(P)

Despite this, a majority (84%) of respondents still felt that existential care was provided in their workplace. A smaller proportion (13%) responded that they did not know if any existential care was provided. For those who answered that existential care was provided, just over half believed that it was given often, while the remaining participants answered that it was provided sometimes. Many commented that it was a natural part of caring for patients.

“In a way we do it all the time, but it’s not so necessarily structured or defined.”(P)

### Responsibility to Offer and Perform Existential Care in the Workplace

This section describes who is responsible for offering existential care (Table 2) and who performs existential care (Table 3).

**Table 2.** Who is Responsible for Existential Care Being Offered? (Several Options Possible).

	Municipal (*n* = 15)	Oncology (*n* = 35)	Palliative (*n* = 27)	Total (*n* = 77)
Occupational therapist	3	5	4	12 (16%)
Manager	6	8	8	22 (29%)
Dietician	1	4	3	8 (10%)
Physiotherapist	4	5	3	12 (16%)
Social worker	4	15	10	29 (38%)
Physician	5	16	9	30 (39%)
Priest/deacon	6	13	9	28 (36%)
Reg nurse	6	18	11	35 (45%)
Assistant nurse	7	12	9	28 (36%)
Other	0	2	3	5 (6%)
All	8	16	20	44 (57%)
Do not know	1	0	1	2 (2%)

**Table 3.** Who Performs Existential Care? (Several Options Possible).

	Municipal (*n* = 15)	Oncology (*n* = 35)	Palliative (*n* = 27)	Total (*n* = 77)
Occupational therapist	3	3	3	9 (12%)
Manager	1	1	3	5 (6%)
Dietician	1	4	2	7 (9%)
Physiotherapist	3	5	3	11 (14%)
Social worker	5	13	9	27 (35%)
Physician	3	10	8	21 (27%)
Priest/deacon	6	13	10	29 (38%)
Reg nurse	5	11	9	25 (32%)
Assistant nurse	5	11	9	25 (32%)
Other	1	3	1	5 (6%)
All	7	17	18	42 (55%)
Do not know	0	4	0	4 (5%)

The majority (57%) of participants felt that all staff categories had a responsibility to ensure that existential care was **offered** in the workplace. That approach was most common among those who worked in specialist palliative care. Six staff categories were most associated with being responsible for providing existential care: reg. nurse, physician, social worker, priest/deacon, assistant nurse and manager. For more details, see Table 2.

In response to the question ‘who is responsible for existential care being offered?’ respondents further commented:

“Everyone who meets a patient in a critical stage is responsible for their behaviour and how to respond and take care of the situation.”(O)

“it is my responsibility as a person who cares for other people...the manager’s responsibility is to ensure there is space to do so.”(P)

When asked who is responsible for existential care being **performed**, the dominant (55%) view among participants was that all staff categories performed existential care regardless of the care context in which they operated. The staff categories most associated with performing existential care were priest/deacon, social worker, reg. nurse, assistant nurse and physician. For more details, see Table 3.

Respondents further commented:

“I believe it is important to work on increasing awareness of existential issues among everyone who interacts with patients.”(P)

“It is not dangerous to talk about existential questions, and it doesn’t always have to be an expert.”(M)

Teamwork was perceived as a strength, as several staff categories could identify the need for existential care and decide if it should be administered by a member of the team or referred to another staff category outside the team.

“Everyone in the team involved with a patient has a responsibility to provide existential care. The manager gives staff the opportunity to work around this.”(O)

Existential care was often associated with conversations, either individual or those held in a nursing context. Existential care also included being with a patient and various forms of touch.

“After all, we are all there with the patient. Sometimes it’s not words that are needed but presence and touch.”(P)

Some described referring existential care to other professional categories when they felt they could not perform this care themselves.

“I consult other professional groups when necessary.”(O)

“We often refer it to a social worker.”(O)

### Dialogue on Existential Issues

More than half (63%) of respondents stated that existential conversations could be experienced as either easy or difficult. Of the remaining participants, 16% answered that it was always easy to talk to patients about existential issues, while 9% expressed it was always difficult. For more details, see Table 4.

**Table 4.** How Do You Experience Talking About Existential Questions?

	Municipal (*n* = 15)	Oncology (*n* = 35)	Palliative (*n* = 27)	Total (*n* = 77)
Easy	5	4	3	12 (16%)
Difficult	2	5	0	7 (9%)
Neither or	0	2	3	5 (6%)
Both	6	22	20	48 (63%)
Do not know	0	1	0	1 (1%)
No answer	2	1	1	4 (5%)

These conversations were perceived differently by participants, which could be due to several factors. Some believed that it could raise existential questions in oneself, which was then perceived as an obstacle.

“It evokes thoughts and feelings in myself.”(O)

Others stated that difficulties depended on how stressed they felt and whether they had time to listen and talk.

Those who described it as easy to talk about these issues felt at ease with the subject matter. At the same time, they stated that a conversation could be held on several levels, which meant that some parts of the conversation were easier than others.

“It depends a bit on the person I am meeting. Some people are easier to talk to.”(P)

Most (70%) participants talked about existential issues with their colleagues, regardless of workplace. A smaller proportion (14%) stated that they did not have such conversations in their staff group. Conversations on existential issues took place as needed, when it was felt that co-workers at the same workplace were facing similar problems and having similar experiences. Other conversations among colleagues about existential issues were organized within the staff group at predetermined times. Regardless of when the conversations took place, it was pointed out that there had to be an open climate to discuss the issues. Some participants expressed that end-of-life care naturally raised existential issues and that these conversations between colleagues were therefore necessary to cope with their work.

“Sometimes you need to talk about difficult things with your colleagues. It is easy to talk to those who are involved in caring for the patient.”(O)

“We have a very open climate in the work group and often care for patients at the end of life.”(M)

“We work with life, the end of life and death. We wouldn’t have made it if we didn’t have open conversations and discussions on that topic.”(P)

### Assessment of Existential Needs and Care

More than half (64%) of respondents stated that existential needs and care were not assessed. Just over a quarter (26%) stated that they did not know whether assessments were made in the workplace. Those who stated that assessments were not made suggested that it could be due to a lack of tools. Some expressed that the Edmonton Symptom Assessment Scale (ESAS) and Integrated Palliative Care Outcome Scale (IPOS) could be used as assessment instruments to identify existential needs.

“Via ESAS, a question about how you feel today sometimes shows a bad number even if symptoms such as pain etc. are not bothersome,”(P)

“The use of IPOS to capture existential needs”(M)

### Written Material to Support Work With Existential Issues and Care

One-fifth (22%) of respondents were familiar with written material to support work with existential issues and care. Some of the participants referred to a brochure on the end of life that describes objective changes to the body when death is near, as well as a textbook for palliative care nurses. Almost half (47%) were not aware of any written material that they could access. Most who responded in this way wanted written material to be procured for the workplace.

“we use certain reference literature based on personal interest, but nothing systematized or adapted to the workplace”(M)

“The brochure ‘Livets sista tid’ [At the end of life] is handed out to relatives, would need more…”(P)

### Need for Support – Education, Guidance and Reflection

A majority of the participants (65%) believed that education in existential issues was needed. It was pointed out that the education needed to be updated, and some stated a desire for education and discussions to be combined. Existential subjects were considered difficult, and it was stated that the knowledge presented needed to be anchored in research as well as how to approach patients.

“Being able to take part in new research and discuss these issues in the working group could lead me to take time to catch up.”(P)

“I think you can never become a complete scholar, so I want to learn more.”(M)

A significant number of respondents (65%) reported a need to receive guidance and reflection in all workplaces. The participants described that it was difficult to work with patients at the end of life, and many expressed a desire for some form of guidance or reflection to cope with their work themselves. Access to guidance varied between workplaces. Within specialized palliative care, respondents were more likely to report having access to guidance. In other workplaces, there were only occasional guidance groups. In the oncology and municipal working groups, it was more common to have reflection groups or no guidance or reflection.

“Sometimes we have patients, loved ones, situations or events that really get to you. For us to move on and be able to handle everything we encounter, we need guidance.”(P)

“Being someone’s lifeline is a heavy burden mentally. It would have been invaluable to have some kind of debriefing.”(O)

“I think it’s important to talk about/share experiences because we sometimes hear difficult things when you are confronted by existential questions.”(M)

## Discussion

This study investigates how professionals caring for people approaching death perceive the existential dimension of care in their current workplace. While the participants in this study worked in different care contexts, there was significant agreement between them with regard to various aspects of existential care.

In the past, existential needs and care were often associated with spirituality and religion (Best et al., 2020; Jeuland et al., 2017). This study was carried out in a Swedish care context, which, similar to the rest of Northern Europe, is considered to be highly secular (Stripp et al., 2023). However, the results of the study illustrate that existential needs frequently arise in all workplaces. This supports the arguments for an updated and broader definition of existential care (Bäckersten et al., 2023; Best et al., 2020; Puchalski et al., 2009). These findings also relate to post-secularism, which argues that a modern, ‘enlightened’ society does not preclude spirituality and religiosity (Habermas, 2006, 2008; Stripp et al., 2023). In this sense, the concept of ‘existential’ encompasses existential orientations, needs and resources of a secular, spiritual and religious kind (Hvidt et al., 2022; Nissen et al., 2021).

To provide existential care, professionals need to recognize what is important to a patient and when existential needs are expressed (O’Brien et al., 2019).

A challenge linked to existential care that many participants expressed was a lack of time, which resulted in them choosing not to address existential questions. The participants described that patients could sense their lack of time and therefore sometimes chose to refrain from discussing their existential needs, as previously reported (Browall et al., 2010; Ozanne et al., 2022; So et al., 2023). The provision of existential care could further be limited or prevented for various reasons. One example was when it raised questions for the professionals themselves, which could affect how they addressed these questions with a patient. This is described by Clare et al. (2022) when professionals’ death anxiety influences their communication with patients. The professionals’ fear of death was not clearly expressed in this study but they referred to thoughts and feelings in themselves as difficulties in those conversations.

The majority of the participants stated that all professional categories have a responsibility to provide existential care. There was a consensus that certain professional categories more than others were responsible for the existential care being offered and that the same professional categories were also the ones who performed that care. These professional categories were mainly reg nurses, social workers, physicians, priests/deacons and assistant nurses.

This responsibility can be defined as being sensitive to and taking such issues seriously. This was particularly prominent among professionals in specialized palliative care and could be linked to the philosophy of palliative care, which has its roots in the hospice movement, and more pronounced teamwork in palliative care compared to other care contexts (Clark, 1999; Fernando & Hughes, 2019). As described above, existential care has traditionally been associated with religious representatives (Jeuland et al., 2017). In accordance with newer and broader definitions and perspectives in the area of existentialism, it appears from this study that all professionals can potentially contribute to existential care (Bäckersten et al., 2023; Best et al., 2020; Francoeur et al., 2016; Hvidt et al., 2022; Kestenbaum et al., 2022; Stenman et al., 2023).

Most participants stated that talking about existential issues with patients could be both easy and difficult. It was not expressed what was difficult or easy but many stated that a conversation could be held on several levels, which meant that some parts of the conversation were easier than others. This could mean that a single conversation can contain different aspects that cause complexity, which may depend on external factors, such as the nature of the subjects discussed and the personalities of those involved. If professionals find it potentially difficult to talk about existential issues, one could assume that the subject may also be difficult for patients (Best et al., 2016).

Similar to other potentially challenging and/or perhaps even taboo subjects, such as sexuality, substance abuse and violence in close relationships, healthcare professionals have a responsibility to demonstrate that it is possible to talk about the subject in question. In the past, existential needs and care were often associated with spirituality and religion (Aygin & Bozdemir, 2019; Haesler et al., 2016). In this context, it can be seen as a welcome finding that a majority of respondents indicated that it is the responsibility of all professionals to offer existential care.

The participants stated that it was common for them to talk about existential issues with their colleagues. This could be when the need arose or in an organized form, such as during guidance and/or group reflection. Both of these were experienced as important and helped participants to face existential questions. It has been shown that guidance and reflection can serve as opportunities for professionals to recognize personal challenges, such as anxieties about death, allowing them to cope with these and practise existential care when similar issues are experienced by patients (Clare et al., 2022; Dawson et al., 2021; Henoch et al., 2015). There seemed to be a consensus among participants that the existential dimension pervades care at the end of life. Meeting patients with life-threatening diseases often raises existential questions even for professionals, which entails a continuous need for reflection (Ozanne et al., 2022; Sand et al., 2018; Udo et al., 2014).

The majority of the participants expressed the need for education on existential issues. Interventions in the form of existential training programmes for professionals have been shown to be beneficial for nurses’ confidence, understanding and management of existential issues (Henoch et al., 2015; Ozanne et al., 2022).

In most workplaces, there was no assessment of existential needs and most participants had no knowledge of specific assessment tools for existential needs. However, there are specific assessment tools developed to identify existential/spiritual needs (Fitchett et al., 2020; Monod et al., 2012) but, to our knowledge, none of these have been translated and validated for the Swedish context.

In some workplaces, professionals use assessment tools that incorporate existential needs, such as IPOS. Another example is the ESAS assessment instrument, which, despite not including questions about existential issues, was used by some participants to assess existential needs. Both these instruments have been translated into Swedish (Beck et al., 2017; Heedman & Strang, 2001).

Written material on existential questions and needs was missing within most of the workplaces. Both information materials and assessment tools were perceived by most to be helpful in their work. Highlighting existing material and continuing to develop professionally oriented knowledge support needs to be considered in the future to facilitate existential care (Kestenbaum et al., 2022; Taylor, 2020).

### Practical Implications

It can be beneficial for all professionals who care for patients with a fatal disease and elderly persons to be able to dare to face the questions called existential themselves. An important result of this study is that the existential dimension is not only about spirituality but that it encompasses the whole life situation. When asking professionals, it is clear that the existential dimension is sometimes hard to pinpoint, and it is often difficult to separate existential care from other care interventions. From this material, and a previous study (Bäckersten et al., 2023) two paths can be distinguished to gain insight into the patients’ existential needs and existential care in general.

The first path starts with professionals identifying clear existential needs, for example patients requesting conversations about the meaning of life, fear of death, loneliness, or a desire to participate in religious activities. The identification of need can lead to some sort of existential care, either by the professional in question or by someone else. Note that identification in itself, i.e. listening and paying attention to a patient’s need, can sometimes serve as existential care.

The second path emerges when professionals who participate in regular care activities come to the realization that the care provided also potentially includes an existential dimension, which in some cases will mean that existential care has occurred. For example, when a professional dresses a wound or has a conversation about continued treatment, they are not only providing physical care or having a conversation about treatment, they are meeting a whole person: “taking care of the body is taking care of the soul” (Bäckersten et al., 2023).

In brief, path one occurs when existential needs are identified, leading to existential care being carried out when appropriate. Path two occurs when care interventions, without a primary focus on existential needs, are carried out and the existential dimension that potentially exists within all aspects of care is identified on a meta-level. Both paths require knowledge and insight, but the latter demands a more advanced analysis of care as a whole and of the existential dimension of care.

## Limitations and Strengths of the Study

This study has both limitations and strengths; The response rate cannot be calculated as information on the number of staff contacted was not obtained. There were also not the same number of respondents in each form of care. The survey language was Swedish, which excluded non-Swedish-speaking people from the study. Males were underrepresented, as were young professionals and professions other than reg. nurses. Swedish society is regarded as highly secular, which could limit transferability outside Scandinavia. The limitations mentioned above may have excluded people who may have had a different perspective on the research questions.

Although the material is limited, the study provides guidance on how staff perceive and assess the existential dimension of care by combining answers to open and closed questions. Choosing a qualitative descriptive approach has the advantage of confining data analysis to preserve what is reported by participants. Most of the professionals had been working in their field for more than ten years and were likely familiar with the subject and terminology raised, which could have influenced the result. A strength of the study is in the coherence of the answers found despite participants being professionals in different care contexts: oncology, municipal care and specialized palliative care.

## Conclusion

This study shows that existential care is generally considered to occur and is described as a natural part of patient care that all professional categories have a responsibility to offer and carry out.

Talking about existential issues with patients can be experienced as both easy and difficult, and most of the participants were able to talk to colleagues about the subject. However, the ability to provide existential care may be limited due to professionals’ lack of time. Participants expressed a need for education and written material regarding existential questions, needs and care, which warrants the development of new guidelines in combination with further research.
